# Neurogenic Bladder in Children with Myelomeningocele

**DOI:** 10.3390/diseases13040117

**Published:** 2025-04-17

**Authors:** Aleksandar Sič, Borko Stojanović, Miroslav Đorđević

**Affiliations:** 1 Faculty of Medicine, University of Belgrade, 11000 Belgrade, Serbia; stojanovic@uromiros.com (B.S.); djordjevic@uromiros.com (M.Đ.); 2 Department of Urology, University Children’s Hospital, 11000 Belgrade, Serbia; 3 Department of Urology, Icahn School of Medicine at Mount Sinai, New York, NY 10029, USA

**Keywords:** myelomeningocele, neurogenic bladder, urodynamics, CIC

## Abstract

Myelomeningocele (MMC), a severe congenital anomaly resulting from neural tube closure defects, poses significant urological challenges necessitating specialized care. This review explores the intricate landscape of MMC within urological practice, advocating for a multidisciplinary approach to optimize patient outcomes. By surveying diverse treatment modalities, this review aims to offer insights into enhancing urological management strategies for MMC and guiding future research directions. At the heart of the conversation lies the pathophysiology of neurogenic bladder dysfunction in children with MMC, with a particular focus on the complexities of diagnosis and the various paradigms guiding urological management. Common complications such as recurrent urinary tract infections are examined alongside non-surgical interventions like intermittent catheterization (CIC) and pharmacotherapy, notably oxybutynin. Additionally, surgical options including botulinum toxin injection and reconstructive procedures are explored to enhance urological outcomes for affected children. By unpacking the complexities of neurogenic bladder dysfunction in MMC, this review emphasizes the imperative of a collaborative, multidisciplinary approach in urological care, ultimately aiming to enhance patient well-being and functional outcomes.

## 1. Introduction

Myelomeningocele (MMC) presents a profound challenge for clinicians due to its severity as a birth defect affecting the central nervous system, yet it remains compatible with life. Arising from a failure in the dorsal fusion of the developing neural tube during embryonic development, this condition manifests between the 3rd and 4th weeks of gestation due to incomplete fusion caudally. This developmental anomaly results in abnormal spinal cord (myelon) development, along with anomalies in its protective covering (meninges) and the vertebral arches during early gestation. The complexity of MMC necessitates specialized care from various medical disciplines, including pediatrics, orthopedics, urology, and neurosurgery, as it impacts various organ systems. In recent decades, substantial advancements have transformed the management of neurogenic bladder in patients with MMC. These include improved pharmacological therapies, the introduction of clean intermittent catheterization (CIC), and enhanced strategies for antibacterial prophylaxis. Despite these improvements, urodynamic studies remain essential for accurate diagnosis and prognosis, particularly because clinical examinations and imaging often lack the sensitivity to detect spinal cord lesions [1,2,3,4,5,6]. Given that neurogenic bladder affects over 90% of children with MMC, it stands as one of the most prevalent and impactful comorbidities in this population, necessitating early identification and individualized management [7]. The location and type of neurological injury determine the specific urinary manifestations observed in each patient [7].

Managing neurogenic lower urinary tract dysfunction in pediatric patients poses a complex challenge for pediatric urologists. Core goals include preserving renal function, minimizing urinary tract infections, achieving continence, and promoting independence as children transition to adulthood. However, the urinary complications associated with neurogenic bladder can significantly impair quality of life and contribute to both physical and psychological distress. Treatment approaches must be tailored to each patient, as the location, severity, and functional implications of neurological damage vary widely—affecting detrusor and sphincter coordination in diverse ways. Consequently, no universal treatment strategy ensures consistent improvement, and frequent medical interventions are often required, further impacting the well-being of affected children [8,9].

This review highlights the critical connection between MMC, neurogenic bladder dysfunction (NBD), and pediatric urology. Given the high prevalence and clinical significance of NBD among children with MMC, urological care becomes a cornerstone of their long-term management. To support a comprehensive and practical approach, this review provides an integrated overview of the anatomical and pathophysiological basis of NBD in MMC, followed by sections discussing clinical presentation, diagnostic evaluation with an emphasis on urodynamic testing, therapeutic strategies, quality-of-life considerations, and future research directions.

## 2. Overview of MMC

Meningomyelocele (MMC) is caused by a combination of genetic predispositions and environmental influences. Although spina bifida can run in families, different forms of the condition within the same family are uncommon, indicating various influencing factors. Environmental agents affecting gestation, such as radiation, pollutants, teratogens, poor nutrition, anti-epileptic medications, inadequate folate intake, and substance use, all increase MMC risk [10]. As mentioned before, MMC arises from defects during primary neurulation, particularly the failure of caudal neural tube closure, leading to exposed neural tissue and potential deterioration from amniotic fluid exposure. Pluripotent cells at the caudal end differentiate to form essential spinal structures. Neural tube defects (NTDs), including spina bifida (SB), encephalocele, and anencephaly, are prevalent congenital anomalies affecting the central nervous system’s formation. Despite preventive measures involving folic acid supplementation, open neural tube defects still affect approximately 0.5 to 1.0 per 1000 fetuses in the United States [11]. Advances in survival rates and reductions in mortality among infants and neonates with SB have been observed. However, mortality rates for live-born infants with SB and MMC remain higher than the national average, with the highest risk factors being premature birth and low birth weight [12,13,14,15,16].

## 3. Neurogenic Bladder (NB)

### 3.1. Anatomy, Physiology, and Pathophysiology

Neurogenic bladder involves a disruption in the complex neurophysiological control of the urinary tract, which normally enables smooth, low-pressure filling and voluntary voiding of urine. The urinary bladder, composed of the detrusor muscle, functions under the coordination of the central nervous system, integrating input from the cortex, brainstem, and spinal cord. The cortex enables voluntary control, while the pontine micturition center (PMC), influenced by cortical input, initiates voiding via spinal reflex arcs. Parasympathetic efferents from the sacral spinal cord (S2–S4) stimulate detrusor contraction through muscarinic receptors, while somatic efferents via the pudendal nerve control the external urethral sphincter. Sympathetic efferents from T11–L2 via the hypogastric nerve contribute inhibitory signals to maintain continence during bladder filling [17,18]. At birth, the assessment of detrusor–external urethral sphincter dyssynergia and bladder emptying efficiency is critical, especially in infants with signs of hydronephrosis or reflux, suggesting prenatal bladder outlet obstruction [18]. In normal physiology, the detrusor, bladder neck, and striated external sphincter function in coordination to allow urine storage at pressures <10–15 cm H_2_O and effective voiding at pressures of 50–80 cm H_2_O in males and 40–65 cm H_2_O in females [19,20]. In neurogenic bladder–sphincter dysfunction (NBSD), damage to central or peripheral neural circuits leads to detrusor overactivity, underactivity, or detrusor–sphincter dyssynergia, disrupting bladder compliance and voiding efficiency. High bladder pressures (>40 cm H_2_O) are associated with decreased glomerular filtration and the risk of upper tract deterioration, including obstructive hydronephrosis and vesicoureteral reflux [21].

### 3.2. Clinical Presentation

Neurogenic bladder, now referred to as “neurogenic lower urinary tract dysfunction”, encompasses a spectrum of urinary storage and voiding disorders resulting from neurological conditions, with dysfunction affecting not only the bladder but also the sphincter and posterior urethra, and typically manifesting as either overactive or underactive bladder syndromes [22].

Neurogenic bladder dysfunction (NBD) arises from direct injury or disease involving the brain, spinal cord, or peripheral nerves. If left untreated, NBD may lead to progressive renal damage, complications such as pressure ulcers, and recurrent urinary tract infections (UTIs), as well as psychosocial consequences stemming from incontinence. The primary goals of treatment include preserving renal function, preventing infections, and halting further deterioration of the urinary tract. While diagnosing NB in children with MMC is generally straightforward, diagnostic challenges may arise in cases of occult spinal dysraphism or central nervous system disorders, where clinico-anatomical correlations can complicate recognition [23,24,25,26].

In children with MMC, neurogenic bladder presents with a constellation of urinary symptoms that significantly affect both health and quality of life. These often include urinary incontinence due to impaired sphincter control, and urinary retention, which increases the risk of bladder overdistension and long-term detrusor dysfunction. Incomplete bladder emptying predisposes these children to recurrent UTIs, commonly presenting with fever, dysuria, and foul-smelling urine. Reduced bladder sensation also contributes to irregular voiding patterns, further exacerbating the risk of bladder overfilling and damage. Moreover, sustained elevated intravesical pressures may result in vesicoureteral reflux (VUR), potentially leading to renal scarring and chronic kidney disease. Proactive and individualized management strategies are essential to prevent complications and enhance quality of life for affected patients [27,28,29,30,31]. Table 1 further illustrates the mentioned urinary issues and their impact on health, providing a visual summary of the conditions described.

### 3.3. Diagnostics

#### 3.3.1. Urodynamic Testing

Urodynamic testing is a critical component in the neuro-urologic evaluation of patients with NB. This method is considered the most objective way to identify lower urinary tract dysfunctions during both the filling/storage and voiding phases [6]. Key urodynamic assessments for urinary function include post-void residual (PVR) volume, urinary flowmetry, bladder cystometrogram (CMG), sphincter electromyography (EMG), Valsalva leak point pressure (LPP), and urethral pressure profile measurements. Noninvasive uroflowmetry, which measures the rate and volume of voiding, is the most frequently used study. Urinary flow rate, determined by the force of detrusor contractions and urethral resistance, can indicate various conditions; high flow rates often suggest neurogenic detrusor overactivity, while low rates may point to low detrusor pressure or urinary outlet obstruction.

A bladder CMG test involves filling the bladder with saline to evaluate its capacity, compliance, sensation, presence of detrusor overactivity, and leak point pressures. Detrusor LPP is defined as the pressure at which leakage occurs without bladder contractions or increased abdominal pressure. High detrusor pressures can be problematic in NGB patients with poor bladder compliance and may indicate a greater risk for upper urinary tract damage. Standard urodynamic studies can be supplemented with stress tests to replicate symptoms and assess urinary leakage. Although there is debate over the universal necessity of urodynamics in NB assessment, it is recommended for patients with spinal cord injury (SCI), advanced multiple sclerosis (MS), or spina bifida who are at significant risk of upper urinary tract damage [32].

#### 3.3.2. Video Urodynamic Studies

Video urodynamics (VUDS) is a diagnostic procedure that integrates urodynamic testing with concurrent imaging of the lower urinary tract. This method assesses both the anatomy and function during different phases of urination, offering crucial information that can influence therapeutic decisions and subsequently improve clinical outcomes for patients. VUDS can comprehensively assess lower and upper urinary tract dysfunction in patients with myelomeningocele (MMC). To improve neurogenic lower urinary tract dysfunction (NLUTD) and prevent complications, minimally invasive therapies or surgical procedures should be recommended for MMC patients with low bladder compliance [33,34].

VUDS is performed by filling the bladder with iodine contrast fluid, allowing detailed imaging during the procedure. A fixed X-ray unit, which can move between 90° and 180°, or a C-arm is used to provide imaging in various views—antero-posterior, lateral, and oblique. Ideally, the urodynamic study (UDS) is conducted with the patient in a natural position, utilizing a radiolucent toilet seat for fluoroscopy during sitting and standing positions to assess urinary incontinence and voiding. However, for patients, particularly those with neurogenic lower urinary tract dysfunction who cannot sit or stand, the UDS is performed in a supine position. The VUDS software combines X-ray images with urodynamic traces, displaying the data on a split screen or through superposition, capturing snapshots at clinically relevant moments like during bladder pressure increases or provocative measures [35].

Despite the limited literature on its effectiveness in neuro-urological patients, UDS, particularly VUDS, remains the standard for evaluating lower urinary tract (LUT) function in this group. UDS is highly effective in managing NLUTD. It provides insights into treatment efficacy and the need for adjustments, prompting reconsideration of follow-up protocols. VUDS offers unique anatomical insights into LUT function, essential for monitoring NLUTD. Detecting adverse VUDS findings is crucial for guiding follow-up in high-risk patients [36].

#### 3.3.3. Voiding Cystourethrography (VCUG)

Voiding cystourethrography (VCUG) remains the gold standard diagnostic method for identifying a range of urinary tract conditions in children, including neurogenic bladder and vesicoureteral reflux [37]. During VCUG, conducted in a specialized fluoroscopic suite, a catheter is delicately inserted into the bladder via the urethra, followed by the injection of contrast dye to visualize both its anatomy and function under fluoroscopy. This essential procedure takes place in two separate phases: the filling phase and the voiding phase. Throughout the filling phase, the bladder undergoes a gradual infusion of contrast dye, monitored by the radiologist to discern any structural abnormalities such as VUR, bladder diverticula, or trabeculation. Once the bladder reaches adequate capacity, the catheter is gently removed, and the patient is prompted to void while the radiologist observes the bladder and urethra under fluoroscopy, marking the onset of the voiding phase. Here, they assess bladder emptying, urethral function, and identify the potential abnormalities, such as bladder outlet obstruction or urethral stricture. Notably, VCUG serves as an important diagnostic tool for identifying structural abnormalities commonly associated with NB, thereby guiding specific treatment strategies and facilitating further prognostic assessment. Furthermore, VCUG plays a huge role in monitoring treatment response over time. Depending on the child’s individual condition, sedation may be administered to ensure comfort and compliance throughout the procedure [38].

Contemporary video urodynamic (VUD) studies enhance traditional voiding cystourethrography (VCUG) by integrating it with urodynamic testing, enabling simultaneous visualization of the urinary tract and measurement of bladder functions, such as sensation, capacity, compliance, and detrusor pressure, through a single double-lumen catheter. VUD is regarded as the gold standard for evaluating lower urinary tract disorders in children, as it assesses bladder and sphincter function while also visualizing bladder morphology and identifying VUR. Due to concerns about radiation exposure in traditional fluoroscopic video urodynamics, a new method using contrast-enhanced voiding urosonography (ceVUS) has been developed. This advanced technique uses second-generation contrast media and harmonic imaging to detect VUR without radiation, offering a safer and highly sensitive alternative for pediatric patients. CeVUS provides significant advantages over conventional methods by better detecting vesicoureteral and intrarenal reflux in conjunction with urodynamic disorders associated with VUR [39].

#### 3.3.4. Vesicoureteral Reflux in NB

Vesicoureteral reflux (VUR) is the most common birth defect affecting children’s urinary tracts. VUR occurs when urine flows backward from the bladder into the ureters and sometimes into the kidneys, instead of being expelled from the body through the urethra. This reflux is due to the dysfunctional bladder dynamics associated with NB, where improper coordination of the bladder and sphincter muscles leads to abnormal pressures within the urinary system [40,41].

In children with neurogenic bladder, VUR can lead to several complications. The backward flow of urine can carry bacteria from the bladder to the kidneys, significantly increasing the risk of UTIs. Recurrent UTIs are a common issue and can lead to renal scarring, which is particularly concerning in a developing child. Over time, renal scarring can result in hypertension and impaired renal function, potentially progressing to chronic kidney disease [42].

Additionally, VUR can sometimes be asymptomatic, making regular monitoring crucial for early detection and management. Diagnosis of VUR typically involves imaging studies such as VCUG, which visualizes the reflux of urine into the ureters and kidneys during bladder filling and voiding. Renal ultrasounds may also be used to assess the kidneys’ size and structure and to detect any signs of damage or scarring [43].

In managing VUR in patients with NB, the primary objective is to maintain low bladder pressures, often resulting in improved bladder compliance. This is crucial because lower bladder pressures can reduce the likelihood of urine refluxing into the ureters and kidneys, thereby protecting the upper urinary tract. Anti-reflux surgery has been shown to significantly improve the management of VUR, particularly in pediatric patients. Recent studies highlight the procedure’s effectiveness in reducing the risks associated with VUR, such as UTIs, renal scarring, and reflux nephropathy [44]. Ureteral reimplantation, including minimally invasive and robotic methods, has demonstrated high success rates and is considered a robust option for preserving upper urinary tract function and preventing renal damage [44,45]. Reflux management and preservation of upper urinary tract function can be effectively achieved through a combination of conservative measures and ureteroneocystostomy procedures in NB cases [24].

Conservative measures can include the use of prophylactic antibiotics to prevent UTIs, bladder training exercises, and medications to relax the bladder. However, in cases where these measures are insufficient, surgical interventions such as ureteroneocystostomy—a procedure to reimplant the ureters into the bladder—can be necessary to prevent reflux and protect kidney function [46,47].

Regular follow-up is essential in the management of VUR. This involves repeated imaging studies and renal function tests to ensure that the management strategy effectively controls the reflux and protects kidney health. The impact of VUR on the quality of life for children with neurogenic bladder can be significant, as recurrent infections and the associated treatments can be burdensome, affecting a child’s physical comfort, emotional well-being, and daily activities. With appropriate and timely intervention, many children can maintain good renal function and enjoy a better quality of life [45].

## 4. Management Strategies

### 4.1. General Principles and Treatment Goals

Management of NB involves addressing substantial medical complexities and significant impacts on quality of life across all age groups. In managing NB in children and adolescents, the primary approach is conservative treatment aimed at preserving the upper urinary tract and maintaining optimal bladder reservoir function [48,49]. Initial urologic evaluations are strongly recommended for all individuals with MMC to assess detrusor–sphincter function and guide subsequent management strategies [25]. These evaluations, complemented by urodynamic studies and selective radiographic imaging, are vital for tailored treatment planning and intervention monitoring. Innovative approaches, such as ultrasound measurement of bladder wall thickness, hold promise in identifying patients at risk of upper urinary tract deterioration. Various management strategies, including maximal anticholinergic therapy, endoscopic interventions, and alternatives to bladder augmentation, exhibit potential efficacy in alleviating bladder dysfunction. Clean intermittent catheterization (CIC) remains a cornerstone intervention for preventing urinary stasis and reducing infection risk, while pharmacotherapy and OnabotulinumtoxinA injections effectively address overactive bladder symptoms and detrusor–sphincter dyssynergia (DESD), respectively. Promising options for managing storage and voiding dysfunction include neuromodulation techniques, while surgical interventions offer reconstructive options for refractory cases [50,51].

### 4.2. Cognitive–Behavioral Treatment and Lifestyle Modifications

Behavioral and conservative measures can be particularly beneficial for patients with neurogenic bladder. These treatments include techniques such as timed voiding, which is especially effective for those with sensory neurogenic bladder [52]. Additionally, habit retraining, verbal prompting, prompted voiding, and pelvic floor physiotherapy are important to manage symptoms in these patients. Lifestyle modifications are also recommended and include reducing fluid intake, caffeine, and soda consumption, losing weight, avoiding acidic fruit juices, and spicy or salty foods. Alkalizing the urine through diet and possibly vitamin D supplementation can also be beneficial [53]. Pelvic floor muscle training, which relies on manual techniques, electrostimulation, and biofeedback, has also proven effective in treating overactive bladder (OAB) [52,53].

### 4.3. Pharmacological Treatment

Antimuscarinic medications are typically the first choice for treating NB in children with SB, but their use is often limited due to side effects. Therefore, oxybutynin is often prescribed for patients with neurogenic bladder conditions characterized by detrusor instability. Approved for use in patients aged 5 and above, this anticholinergic drug acts as a muscle relaxant within the bladder. Its active metabolite, N-desethyloxybutynin, competitively inhibits postganglionic muscarinic 1, 2, and 3 receptors, blocking the muscarinic effect of acetylcholine. This action results in the relaxation of bladder smooth muscles, thereby increasing bladder capacity and reducing urinary urgency and frequency [54,55,56].

Furthermore, mirabegron has shown efficacy in managing NB symptoms, particularly in patients with storage symptoms, when antimuscarinic medications fail to improve the condition [57]. Despite this, there is still insufficient evidence to recommend mirabegron as an initial treatment option for NB. Combining antimuscarinic drugs could benefit patients with NB that does not respond to monotherapy. However, antimuscarinic agents are linked to adverse side effects. For patients experiencing urgency urinary incontinence that does not respond to antimuscarinic therapy, recommendations from both the American Urological Association (AUA) and the European Association of Urology (EAU) suggest considering intravesical injection of botulinum toxin A as a treatment option [57,58,59]. Intradetrusor botulinum toxin (BoTA) injection has transformed the management of NB, offering patients a safe, effective, and cost-efficient approach to alleviate bladder dysfunction, preserve renal function, and reduce the necessity for invasive surgical procedures. Similarly, intravesical botulinum toxin (BoNT) injection effectively reduces urgency and urinary incontinence by temporarily inhibiting detrusor muscle contraction. It achieves this by blocking the release of acetylcholine (Ach) from both preganglionic and postganglionic nerves, as well as ATP release from purinergic efferent nerves in the detrusor muscle. Additionally, BoNT-A injection decreases urothelial ATP release and increases nitric oxide (NO) release from the urothelium in afferent nerves. While FDA approval is limited to neurogenic detrusor overactivity and refractory overactive bladder, emerging clinical trials suggest benefits for various functional urological disorders. Patient selection and urodynamic evaluation for diagnosis confirmation are essential for optimizing BoNT-A treatment outcomes. Still, botulinum toxin carries the risk of high urinary residuals, urinary tract infection, and the need to self-catheterize [60,61,62].

### 4.4. Minimally Invasive Treatment

Intermittent catheterization (IC) presents the main method for managing urinary drainage in patients with neurogenic lower urinary tract dysfunction. Over the past two decades, significant advancements have occurred in catheter technology, reflecting an evolution in available tools for IC. Clean intermittent catheterization (CIC) is widely recognized as the gold standard for bladder management due to its low complication rate. Early initiation of CIC, along with pharmacotherapy, has shown superior outcomes compared to an expectant approach. Both the European Association of Urology (EAU) and the European Society for Pediatric Urology (ESPU) strongly suggest the prompt initiation of clean intermittent catheterization (CIC), ideally soon after birth. This approach is linked with reduced renal complications and decreased future need for bladder augmentation. Moreover, it tends to be better received by both parents and patients, among various other advantages [63,64,65,66].

### 4.5. Clean Intermittent Catheterization (CIC)

Over the last 50 years, clean intermittent catheterization (CIC) has become a standard practice for individuals with neurogenic bladder, significantly contributing to renal preservation [67].

CIC is performed by regularly inserting a catheter, a thin flexible tube, into the urethra to drain urine, thereby preventing urinary tract infections (UTIs), bladder overdistention, and potential kidney damage. For children who find urethral catheterization challenging or painful, a surgically created catheterizable channel, such as a Mitrofanoff stoma, may be considered. This channel is constructed using a piece of the child’s appendix or bowel, creating a passage from the bladder to an easily accessible abdominal stoma, through which a catheter can be inserted. The appropriate age to start CIC varies, but it typically begins when the child is around 3 to 5 years old, depending on their cognitive and physical development. At this age, children can start learning the process with assistance from their caregivers, aiming to foster independence in managing their bladder function as they grow older. Early initiation of CIC, combined with proper education and support, is crucial for long-term bladder health and overall quality of life for these children [67,68,69,70,71].

### 4.6. Surgical Treatments

In cases where intermittent catheterization via the urethra or a continent stoma is not feasible, management with an incontinent stoma becomes necessary. Surgical options such as cutaneous ureterostomy, vesicostomy, ileal conduit, and ileovesicostomy are available and used, with vesicostomy commonly used as a temporary measure in pediatric neurogenic populations [72].

#### 4.6.1. Urethral Dilation

Urethral dilation has been shown to effectively reduce detrusor leak point pressure in children with NB, helping address upper tract dilatation and VUR. A recent study highlights the benefits of this procedure, noting significant improvements in 68% of cases and minimal need for further surgical interventions. This supports urethral dilation as a viable option in managing NB in pediatric patients, emphasizing its role in preventing further complications and preserving upper urinary tract function [73].

#### 4.6.2. Cystoscopic Injection of Botulinum Neurotoxin (BoNT)

Administering botulinum toxin (BoNT) injections while the patient is awake is a viable option for pediatric patients with NB who undergo clean intermittent catheterization (CIC). Offering awake BoNT injections as an early intervention, it enhances the practicality of BoNT as a valuable tool in the comprehensive management of NB in pediatric patients [74].

Patients may receive either general anesthesia or sedation during the procedure, as botulinum toxin is administered directly into the detrusor muscle using cystoscopy. A dose of 200 units of botulinum toxin is generally recommended for cases of neurogenic detrusor overactivity, with a higher dose of 300 units considered depending on the physician’s judgment. Procedural side effects include temporary urinary tract infections and hematuria. Instances of incomplete bladder emptying and urinary retention may occur, but systemic symptoms are exceedingly rare due to the minimal dosage administered. Botulinum toxin injection offers positive changes in urodynamic measurements and clinical symptoms, lasting around 9 months. Furthermore, botulinum toxin treatment appears to be highly tolerable, with minimal side effects reported [75,76,77,78].

#### 4.6.3. Sacral Neuromodulation 

Sacral neuromodulation (SNM) is considered a minimally invasive and innovative approach for addressing NB. However, its efficacy and safety are still being explored, and current research remains in the preliminary stages, lacking clarity on its effectiveness and safety profile [79].

Sacral neuromodulation is conducted in stages, beginning with the percutaneous insertion of a quadripolar lead into the S3 foramen under fluoroscopic guidance. This lead is then connected to an external pulse generator. If patients show a significant improvement of over 50% in urinary symptoms, they may proceed to have an implantable pulse generator installed. Complications arising from SNM have been extensively documented, but with the majority being just minor adverse events [80,81].

#### 4.6.4. Bladder Augmentation—Nterocystoplasty

Augmentation cystoplasty (AC) has long been a standard treatment for addressing issues such as low bladder capacity, poor compliance, or unresponsive OAB. Despite the emergence of alternative therapies such as intravesical botulinum toxin and sacral neuromodulation for detrusor overactivity, the demand for AC in these cases has diminished. Nonetheless, AC remains pivotal in pediatric and renal transplant care, as well as for managing refractory OAB. Moreover, advancements in surgical techniques have introduced laparoscopic and robotic approaches to AC. When conservative measures, medications, and less invasive interventions have proved inadequate, AC remains a viable option for both neurogenic and non-NBD, often having high patient satisfaction rates, as it helps increase bladder capacity and decrease complications in the upper urinary tract [82,83].

AC involves utilizing various segments of the bowel to increase bladder capacity. Enterocystoplasty serves as a broad term indicating the use of a bowel segment for this purpose, with specific terminologies applied based on the specific bowel segment harvested. The most commonly utilized bowel segment for AC is a detubularized patch of ileum [84].

**Figure 1:**
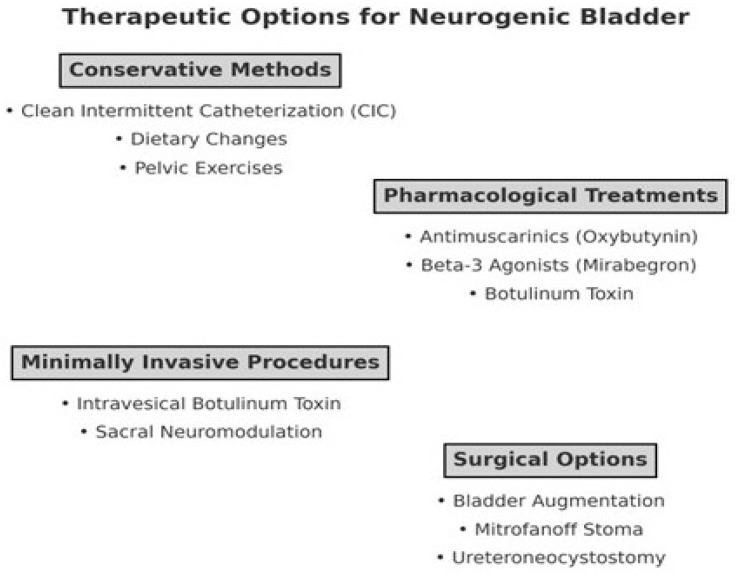
Summarized treatment options for the treatment of NB.

However, certain conditions pose contraindications for AC, including Crohn’s disease, congenital anomalies like cloacal exstrophy, radiotherapy-induced enteritis, extensive bowel resections resulting in short bowel syndrome, and malignant bladder disease. These factors warrant careful consideration in treatment planning. Additionally, AC carries a risk of developing malignancies [84,85]. Figure 1 shows summarized therapeutic options for the treatment of NB.

### 4.7. Cost-Effectiveness Considerations

Evaluating the cost-effectiveness of various treatment modalities for neurogenic bladder in children with MMC is essential, particularly in healthcare systems with limited resources. According to the EAU/ESPU guidelines, conservative strategies such as the early initiation of CIC and antimuscarinic pharmacotherapy are associated with reduced risk of upper urinary tract deterioration and a lower likelihood of requiring surgical interventions later in life. These approaches are not only clinically effective but also represent economically sustainable options. In contrast, while interventions like intradetrusor botulinum toxin injections and sacral neuromodulation offer promising results, their high costs and potential need for repeated procedures may limit accessibility. Augmentation cystoplasty, although effective in refractory cases, entails significant long-term healthcare costs due to complications, ongoing surveillance, and the risk of malignancy. Thus, tailoring treatment plans to balance clinical benefit with economic feasibility remains a critical component of multidisciplinary care for patients with MMC-related neurogenic bladder [65].

## 5. Monitoring Neuro-Urological Dysfunctions

Urodynamic testing plays a central role in the evaluation and long-term management of neurogenic bladder. It provides detailed insights into bladder compliance, capacity, detrusor overactivity, and sphincter coordination—parameters that are essential for the early detection of potentially harmful bladder behavior. These findings are often more sensitive than clinical symptoms or imaging, especially in pediatric patients with MMC, and can directly influence therapeutic strategies aimed at preventing irreversible renal damage. Regular urodynamic surveillance is therefore not only diagnostic but also prognostic, guiding both immediate interventions and long-term care planning [36,86,87].

For patients with NB, regular urodynamic testing is crucial for assessing bladder function and guiding further treatment decisions. However, the intervals between these tests may result in bladder decompensation or hydronephrosis. To address this issue, cystomanometer (CM) and cystoelastometer (CEM) have been developed for home bladder pressure monitoring. The handheld CM measures opening bladder pressure and timestamps it, while the CEM actively evacuates urine and records the volume evacuated. Both devices transmit data wirelessly to a smartphone, where a dedicated application securely stores, displays, and sends the data to a hospital server. Additionally, patients with spina bifida, particularly those with MMC, require regular kidney ultrasound monitoring annually or biennially, to ensure ongoing renal health assessment. High-risk individuals also include those with adverse urodynamic findings such as impaired bladder compliance, detrusor–sphincter dyssynergia, or vesicoureteral reflux [88,89].

In addition to functional evaluation and risk stratification, the comprehensive management of neurogenic bladder should integrate strategies aimed at improving quality of life (QOL) for both patients and their families. These strategies include promoting adherence to bladder management protocols such as clean intermittent catheterization (CIC) through individualized education, addressing psychosocial stressors, and ensuring access to mental health services. Multidisciplinary follow-up—encompassing pediatric urologists, nurses, psychologists, and social workers—can significantly reduce caregiver burden, foster patient autonomy, and enhance long-term outcomes. Furthermore, empowering families through consistent education, community-based support, and structured transitional care planning is essential for maintaining QOL throughout childhood and adolescence [90].

## 6. Conclusions

Neurogenic bladder in children with MMC represents a complex and multifactorial condition that demands early recognition, comprehensive evaluation, and individualized long-term management. The interplay between neuroanatomical abnormalities and urological dysfunction results in variable clinical presentations, making standardized care challenging. Over the past few decades, advancements in diagnostic techniques such as urodynamic testing and imaging modalities, along with refined surgical and pharmacological interventions, have significantly improved outcomes and preserved renal function in affected children.

Despite this progress, several areas require further research. Long-term prospective studies are needed to evaluate and compare the effectiveness of conservative versus surgical approaches in preserving bladder and kidney function across different stages of development. There is also a growing need to explore novel neuromodulation techniques and bioengineering strategies aimed at enhancing bladder control while minimizing complications. Moreover, future research should focus on personalized treatment protocols guided by neuroimaging, genetic, or urodynamic biomarkers that could improve early stratification and targeted therapy.

Overall, a multidisciplinary and evidence-based approach remains essential in optimizing outcomes for children with MMC-related neurogenic bladder. Integrating emerging technologies and personalized treatment strategies—guided by urodynamic, genetic, and imaging biomarkers—holds the promise of more precise, patient-centered care. Furthermore, prioritizing early conservative interventions such as CIC and antimuscarinic therapy not only improves clinical outcomes but also represents a cost-effective strategy in preserving renal function and delaying invasive treatments. As healthcare systems increasingly face resource constraints, future research must also incorporate health economic evaluations to support rational, equitable, and sustainable decision-making in the long-term management of MMC-associated neurogenic bladder.

## Figures and Tables

**Table 1:** Summary of bladder and sphincter dysfunction in pediatric NB due to MMC.

Combination	Functional Description	Urodynamic Findings	Health Impacts
Overactive Sphincter and Overactive Bladder	Both are hyperactive; often associated with urgency and pain	Increased intravesical pressure	Urinary incontinence, pain
Overactive Sphincter and Underactive Bladder	Sphincter is hyperactive, bladder is hypoactive	Evident urine retention	Issues with bladder emptying
Underactive Sphincter and Overactive Bladder	Sphincter is hypoactive, bladder is hyperactive	Increased intravesical pressure and reflux	Urinary incontinence, frequent urination
Underactive Sphincter and Underactive Bladder	Both are hypoactive; may lead to excessive retention	Decreased intravesical pressure	Problems with emptying and incontinence
